# Korean Sibling Caregivers of Individuals Diagnosed with Schizophrenia

**DOI:** 10.9741/23736658.1066

**Published:** 2017

**Authors:** Mijung Park, Kwang-Ja Lee

**Affiliations:** a University of California, San Francisco, USA; b Ewah Womans University, Republic of Korea

**Keywords:** sibling, sibling caregiving, brothers, sisters, family, family caregiving, schizophrenia, Colaizzi, qualitative research

## Abstract

Siblings of individuals diagnosed with schizophrenia are an important source of family caregiving. Unfortunately, limited information is available about sibling caregivers because existing studies have focused on other family relationships such as parents, spouses, and children. To fill the knowledge gap, the purpose of this study is to describe Korean sibling caregivers’ experience with individuals diagnosed with schizophrenia.

Guided by Colaizzi’s descriptive phenomenological methodology, we conducted in-depth, semi-structured, face-to-face interviews with eight individuals who have a sibling (1) diagnosed with schizophrenia and (2) hospitalized in an inpatient psychiatric unit. We discerned six key themes: sorrow, burnout, shame, different perspectives in life, acceptance, and responsibility. We categorized these themes into three groups: suffering, hope, and responsibility and obligation.

Sibling caregivers of individuals with schizophrenia experience a mixture of several emotions. Participants loved their brother or sister with schizophrenia, but at the same time they felt shame and fear. While they were burdened by the responsibilities of caregiving, they remained loyal to their sibling with schizophrenia, continuing to help their siblings reach their full potential. Although participants were confused about the symptoms of schizophrenia, they were committed to learning more about the illness.

Because we conducted the current study in Korea, the findings of this study may be unique to Korea culture. Further studies are needed to compare and contrast nuanced differences in sibling caregivers’ experience among different cultural groups.

Schizophrenia is a chronic brain disease in which the patient loses contact with reality, affecting people of all races, cultures, and social classes. Schizophrenia is associated with psychotic symptoms, social withdrawal, and unusual behavior. About seven individuals per 1,000 of the global population has been diagnosed with schizophrenia at one point of their life (McGrath, Saha, Chant, & Welham, 2008).

Schizophrenia has profound short-term and long-term impacts on patients and their loved ones, leading to impairment in cognitive, social, and occupational functions (Aleman, Hijman, de Haan, & Kahn, 1999; Bell & Bryson, 2001; Dodell-Feder, Tully, & Hooker, 2015; Lay, Blanz, Hartmann, & Schmidt, 2000). For caregivers, caring for a family member diagnosed with schizophrenia can lead to emotional, financial, social, and physical burdens. Family caregivers often experience fear and embarrassment about symptoms, uncertainty about the course of the disease, lack of social support, and stigma (Awad & Voruganti, 2008).

The sibling relationship is a unique bond that lasts a lifetime. Siblings share overlapping developmental stages and influence each other’s developmental processes. While parents and spouses are the most common primary family caregivers, siblings frequently share caregiving responsibilities (Horwitz, 1993). There are two reasons why sibling caregivers may play more important roles in individuals with schizophrenia than in those with other conditions. First, because of the high emphasis on deinstitutionalization and the lack of community-based services, family caregivers often fill the gaps in mental health care systems. Secondly, the life expectancy for individuals with chronic mental illness, such as schizophrenia, has improved over the past decades (Laursen, 2011). Because individuals diagnosed with schizophrenia frequently stay single and do not have children, they may have to rely on their sibling after their parents become too old to care for them or die.

Unfortunately, among many social relationships in the context of family caregiving, sibling caregivers are least studied. There is little known about how people are affected by their sibling’s schizophrenia. To fill this gap, the purpose of this study was to describe what it is like to care for a sibling diagnosed with schizophrenia from the perspective of their healthy sibling.

## Method

Colaizzi’s descriptive phenomenological investigation (1973) guided the design, data collection and analyses, and reporting of this study. We followed the seven sequential steps described by Colaizzi: interviews, extraction of significant statements, formulating meaning, grouping of the formulated meanings, naming themes, grouping themes into clusters, and exhaustive description of the phenomena. We describe each step in the analyses section in more detail. We conducted and analyzed all interviews in Korean. The quotes from participants presented in subsequent sections have been translated into English.

### Inclusion and Exclusion Criteria and Recruitment Strategies

We used purposeful sampling to recruit participants. To be included in the study, Korean participants had to 1) be aged 20 years and over, 2) have a sibling diagnosed with schizophrenia for two years or more, 3) reside with or has at least weekly contact with the sibling with schizophrenia, 4) agree to participate in the study, and 5) the sibling with schizophrenia had to be hospitalized in an inpatient psychiatric unit at the time of recruitment. Exclusion criteria included having been diagnosed with schizophrenia.

We posted flyers in the inpatient psychiatric unit located in Seoul, Korea. When a potential participant contacted the research team, the first author (MP) met with the potential participant and obtained informed consent. We also used snowball sampling technique to identify more potential participants. Ewah Womans University Institutional Review Board approved this study.

### Data Collection

The primary source of data was face-to-face, in-depth interviews. MP conducted open-ended and minimally structured interviews with participants. Interviews focused on participants’ experience with caring for their sibling diagnosed with schizophrenia. Minimal prompts were used during the interview to let the story unfold; follow-up questions included using narrative and reflective questions (Benner, Tanner, & Chesla, 1996). Each interview was recorded with the participant’s consent and transcribed verbatim immediately after completion.

In addition to face-to-face interviews, data included field notes and memos. Field notes—written during and after interviews—provided details about the context and the flow of each interview, the interviewer’s initial interpretations of what was said, and the reflections about the interview. Memos—written during analyses—documented discussions among research team members and the process of analytic decisions.

We considered data saturated after the seventh interview. An additional interview was conducted to confirm the saturation. Each interview lasted between 45 minutes and one hour.

### Analyses

Data analyses were conducted in tandem with data collection, allowing the research team to further explore emerging themes in subsequent interviews. As mentioned earlier, we followed the steps proposed by Colaizzi (1978).

First, research team members read each transcribed interview multiple times to gain a general sense of the whole content. Second, from each transcribed interview, we identified and extracted significant statements and phrases. These were sentences and phrases directly related to the phenomenon of interest (i.e., experience with a brother or a sister diagnosed with schizophrenia). As the number of interviews increased, we identified the same or similar statements across several interviews and selected the most representative statement, as advocated by Colaizzi. The following quote, from a male participant whose younger sister was diagnosed with schizophrenia, was an example of a significant statement.

Participant (P)3: I think I have to enjoy life and do my best in everything. In the cases where my sister does not get well, I have to take care of her after my mother and father pass away. I am pretty sure that I will get some help from my extended family. But I am her only brother. I feel like that I am the only person who can take care of her anyhow.

Third, we identified the underlying meanings of each significant statement and phrases. Colaizzi referred to this step as “formulated meaning.” During a team meeting, research team members validated both significant statements and their formulate meanings.

Fourth, we grouped formulated meanings based on common themes across the interviews. We sorted these themes into theme clusters and highlighted commonalities among themes. For example, the theme “embarrassed” and “feeling stigmatized” were included in the theme cluster “stigma.” The aggregated clusters of themes were discussed and validated during the research team meeting. Finally, we organized theme clusters again into three theme categories: suffering, hope, and responsibility and obligation.

## Results

Among the eight participants, three were male and five were female. Four participants were in their 20s, three in their 30s, and one participant was in his 40s. While all participants had a sibling diagnosed with schizophrenia, the chronicity of the sibling’s illness varied. Three participants had a sibling who had more than five inpatient psychiatric hospitalizations, while one participant was experiencing a brother’s first psychiatric inpatient hospitalization.

Table 1 describes processes of phenomenological reduction. We extracted a total of 146 unique significant statements. These statements generated 69 formulated meanings, which we categorized into 13 themes. We then sorted them into six theme clusters: sorrow, burnout, shame, different perspectives in life, acceptance, and responsibility. Finally, three theme clusters emerged: suffering, hope, and responsibility and obligation.

### Suffering

The theme category “suffering” describes the negative experience associated with having a brother or a sister with schizophrenia: sorrow and sadness for losing their once healthy sibling, feeling confused about the illness, difficulty coping with worsening symptoms, experiencing burnout by repeated hospitalizations, and stigma and shame. All participants reported experiencing difficulty in understanding their sibling’s illness, symptoms, and behaviors. They felt helpless and frustrated because of a lack of understanding.

P2: It was as if she became different person. You know. She has such calm and pretty eyes. But when she became sick, you can tell by her eye. Her eyes are full of anger. I am very scared and can’t even look at her eyes when it happens. It seems like that she is actually a different person. She told us that we are not her family. Sometimes, I think that she really does not recognize me. If we meet on the street, she may not recognize me. It is so sad because she’s my baby sister.

Participants were aware of the stigma associated with schizophrenia and worried about other people’s attitude towards their sibling’s mental illness. At the same time, they internalized such stigma, feeing shame about having a brother or a sister with schizophrenia. One participant described how he tried to keep his sister from attending his wedding because he was afraid of how other people might react to her symptoms:

P2: My sister was not able to make it to my wedding.

Interviewer: Oh... Was she in the hospital?

P2: No. We decide have her stay home. You know, she is quite unpredictable. You don’t know what is going to happen when she’s there. She maybe yell or scream. She’s very sensitive. Wedding party is loud. So, we thought it might be better for her not to come. My in-laws knew about my sister. But they’ve never met her to this day. I know that it is unfair but you know how people are.

### Hope

The theme category “hope” refers to positive impacts and gains from caring for a brother or a sister with schizophrenia. Participants tried to focus on strengths of their sibling with schizophrenia: what they can do and what they admired about them. Participants also described the positive influence of having a sibling with schizophrenia: compassion toward others, deepened sibling bonds, and personal growth.

P1: I hope that he (patient) can get married and have children. He will make such a great father. When he is not in the hospital, we go to the grocery shopping together. My mother give us a list. He chooses the best fruit. He is very good with my children too. He is a great uncle. I wish he meet a girl who can understand his illness.

Having a sibling with schizophrenia helped participants gain personal and experiential knowledge about schizophrenia. They developed different perspectives about life from caring for their brother or sister with schizophrenia. Through these new perspectives, feelings of hope for the future with their sibling with schizophrenia have developed.

Hope also included staying positive, relying on God or being spiritual, and having faith that their sibling would get better. Participants reported that the quantity and the quality of the relationship with other family members improved after learning about their sibling’s mental illness.

P8: I sometimes think that God sent us the illness to teach us. Guide us. Now I appreciate my family and my life much more. I cherish my husband and children. Sometimes I get frustrated with my children, but then I think about P [the patient], then I feel like I can calm down faster. You never know what’s going to happen in life. Once you realize that, every moment becomes a gift from God.

### Responsibility and Obligation

The theme category “responsibility and obligation” describes when participants accepted the chronicity of schizophrenia and were willing to step up as the main caregiver when necessary. For participants, caring for their sibling with schizophrenia meant balancing their own life, work and relationships; or putting their lives on hold while providing a stable and secure environment for their sibling.

P4: It has been about 15 years since my sister was first hospitalized. You know it is not cheap to have your family in the hospital. Most of the year, she used up the maximum benefit days. Sometimes, social workers help us to get discount on the hospital bills. At first, we did not even know that there was a limit on how long you can stay in the hospital. But I did not want to send her to the state hospital. I heard horrible stories. That is the place for those who were abandoned by their own family. My sister has a family. There is no way she will go there. My family has been suffering because of M [the patient]’s illness. I am not saying that it is her fault. But its impact on us was quite significant.

Participants accepted that schizophrenia is a chronic illness and were prepared to step up as the main caregiver when their parents pass away. Such an acceptance of future responsibility led participants to have an increased interest in learning about schizophrenia and planning for the future in more practical terms.

P7: At first, I thought that it [schizophrenia] would be like cold or broken leg. If he rests in the hospital for a while and get a good medication, he will be healthy again. But now I think I have to accept that he will not going to be exactly the same. And our family need to start planning what to do about it. My mom and dad are getting old. When they are passed away, then I am the next in line. I need to know a lot about this illness. We actually talked about what to do with money when my mom and dad passed away. How to manage money well so that we can help him to afford new treatment or medication.

## Discussion

The central finding of this study is that a person’s schizophrenia impacts his or her siblings emotionally, socially, and psychologically. Sibling caregivers of individuals with schizophrenia experience a mixture of multiple emotions. Participants loved their brother or sister with schizophrenia, but at the same time they felt shame and fear. While the responsibilities of caregiving burdened participants, they remained loyal to their sibling with schizophrenia, continuing to help their siblings reach their full potential. Although participants were confused about the symptoms of schizophrenia, they were committed to learning more about the illness.

Because we conducted this study in Korea, we anticipated that the participants’ experiences might be unique to the culture and the mental health care systems in Korea. However, we found that findings of past studies—while limited in number—are generally consonant with findings of the current study (Barnable, Gaudine, Bennett, & Meadus, 2006; Gerace, Camilleri, & Ayres, 1993; Kristoffersen & Mustard, 2000; Smith, Greenberg, & Mailick Seltzer, 2007; Stålberg, Ekerwald, & Hultman, 2004).

Existing qualitative studies on siblings of individuals diagnosed with schizophrenia have been conducted in Canada (Barnable et al., 2006), Norway (Kristoffersen & Mustard, 2000), Sweden (Stålberg et al., 2004), and the United States (Gerace et al., 1993). General themes that emerged from this study were similar to the other studies in the literature: sibling bonds, love and sorrow, anger and shame, and coping and caregiving. These common themes also emerged from the data in this study. There are, however, two noteworthy variations in findings between existing qualitative studies and the current one. First, the fear of possible heredity of schizophrenia was a notable theme in Stålberg et al.’s (2004) and Barnable et al.’s (2006) studies. Such a theme did not emerge in this study because participants in the current study did not voice such concern. Because each study used a different interview guide, exploring dissimilar aspects of the sibling caregiver experience, this may explain the variations in emerging themes. Second, the sense of responsibility and obligation to take care of the brother or sister with schizophrenia was a major theme in the current study. However, it was not explicit in the studies conducted with Western participants. Differences in expectation for the sibling relationship between Eastern and Western cultures may explain this variation. The mandate for caring for an ill sibling is consistent with the Confucian notion of family, a predominant philosophical underpinning of Asian society (Park, 2012; Park & Chesla, 2007). Further studies are needed to compare and contrast such nuanced variations in sibling caregivers’ experience among different cultural groups.

A limited number of quantitative studies have examined the siblings of individuals with schizophrenia (Friedrich, Lively, & Rubenstein, 2008; Smith & Greenberg, 2007; 2007; Smith, Greenberg, Sciortino, Sandoval, & Lukens, 2016). Collectively, the authors concluded that (1) more than three fourths of siblings of individuals with schizophrenia reported that they anticipate to care for their brother or sister with schizophrenia in the future, and (2) the quality of the sibling relationship are closely related to the healthy sibling’s willingness to engage future caregiver roles for their brother and sister with schizophrenia. Therefore, developing interventions to improve the quality of the sibling relationship may be beneficial to both individuals diagnosed with schizophrenia and their sibling caregivers.

Participants in the current study reported feeling confused about the symptoms of schizophrenia. The difficulties in managing schizophrenia symptoms often lead to increased burden. Therefore, educational programs for sibling caregivers of individuals with schizophrenia could prevent or lower the sibling caregiver burden. Moreover, participants reported experiencing complex negative emotions such as sadness over losing a healthy sibling, stigma, and shame. Therefore, support programs to help sibling caregivers cope with such negative emotions are needed. Studies have shown that the well-being of individuals with schizophrenia and that of their sibling caregivers are connected (Friedrich et al., 2008). Therefore, educational and support programs for sibling caregivers have the potential to improve the well-being of individuals with schizophrenia.

Research interests in family caregivers have steadily increased over the past several decades. Yet, sibling caregivers have not received the attention they deserve. The current study addresses this knowledge gap by directly examining sibling caregiver’s lived experience with individuals diagnosed with schizophrenia. Future studies are needed to move the field of family caregiving research and practice forward. First, we need more information about how different family relations (e.g., sibling vs. parent) impact the way we cope with illness in the family. Second, it is largely unknown how cultural variations in social expectations surrounding sibling relationships impact sibling caregiving practices among a multicultural society. Furthermore, how acculturation impacts sibling relationships and sibling caregiving is rarely examined. Therefore, we need more research on the sibling relationship in the context of caregiving among the multicultural families. Third, we need more research to identify interventions to support sibling caregivers of individuals with schizophrenia.

We acknowledge that, because participants in this study were recruited from an inpatient psychiatric unit, their experience may be different from those who have not experienced inpatient hospitalization of their sibling. Therefore, future studies need to explore perspectives of sibling caregivers of individuals diagnosed with schizophrenia residing in the community.

## Conclusions

We found that healthy siblings of individuals with schizophrenia experience complex and layered emotions. Findings of this study, conducted in Korea, are similar to those of existing studies conducted in other countries, suggesting that such complex experiences may be common among healthy siblings caring for those with schizophrenia. Further studies are needed to examine cultural variations and similarities in sibling caregivers’ experience across diverse populations.

## Figures and Tables

**Table 1:** Schematic Process Phenomenological Reduction

Formulated Meaning	Theme Clusters	Theme Categories
Sorrow	Sorrow	Suffering
Regret		
Worry		
Feeling tense		
Feeling tired	Burnout	
Anxious		
Embarrassed	Stigma	
Being Stigmatized		

Hope	Hope	Hope
New worldview	Different perspectives	
Increased understanding about schizophrenia		
Improved understanding among family members		

Feeling obligated for taking care of the sibling	Accepting responsibilities	Responsibility and obligation
Preparing for the future as a caregiver		
